# The Utah Manipulation and Locomotion of Large Objects (MeLLO) Data Library

**DOI:** 10.3390/bioengineering12030317

**Published:** 2025-03-19

**Authors:** Nathaniel G. Luttmer, Nathan I. Baum, Josue Flores-Gonzalez, John M. Hollerbach, Mark A. Minor

**Affiliations:** University of Utah Robotics Center, John and Marcia Price College of Engineering, University of Utah, Salt Lake City, UT 84112, USA

**Keywords:** databases, kinematics, kinetics, dynamics, human–object interaction, haptics, legged locomotion, data models

## Abstract

The purpose of this paper is to provide researchers with a description of a data library representing human interaction with medium- to large-sized objects in everyday life. The library includes motion capture data characterizing human and object motion, as well as data for characterizing haptic interaction with the object via force and torque measurements via a load cell and inertial measurement unit (IMU) readings of the object accelerations. Objects include a box, luggage, briefcase, walker, shopping cart, wheelbarrow, and door. The data collected includes multiple types of interactions with each object, such as manipulating the object and walking while interacting with the object (e.g., pulling, pushing, carrying, operating, etc.). Data processing techniques for synchronizing data, deriving human biomechanics, and segmenting trials are presented. Examples of how the data in the library can be manipulated and processed further are provided. This includes combining ten wheelbarrow lifts of one subject together and analyzing the knee motion, object acceleration, and load cell readings (force and torque) with mean trajectories and standard deviations of the trajectories. From there, the range of motion can be extracted, such as for the hip, knee, and ankle joint minimum angles, maximum angles, and range of motion. A comparison of walking with and without a wheelbarrow is presented using spatiotemporal parameters and cyclograms to demonstrate their differences. The database is available on AddBiomechanics, SimTK, and GitHub.

## 1. Introduction

Humans interact with many different objects throughout their daily activities. Some examples include carrying a briefcase to work, pushing a cart while shopping for groceries, working in the garden with a wheelbarrow, and lifting and moving boxes. Likewise, humans who have trouble walking use assistive devices to help them walk to places they need to go. The way humans interact with these different objects varies in many ways. Some objects can be interacted with using only one hand, and some need to be interacted with using both hands due to the size and shape of the object. The area where humans place their hands to interact with the object is also different given the shape and size of different objects. Humans approach and interact with these objects differently, and the biomechanics moving these objects also varies. This paper presents a library of data characterizing humans interacting with several objects from daily life, which will be used to develop a better understanding of human motion, object motion, and haptic interaction between humans and objects in future work.

## 2. Motivation

Libraries of data characterizing human manipulation and interaction help scientists and engineers characterize how humans interact with those objects. Such data provide a foundation for ergonomists, biomechanicians, roboticists, and virtual reality and gaming engineers seeking to characterize human biomechanics and develop new devices and technology to simulate those interactions. Understanding the forces, torques, and dynamics produced on objects when a human interacts with them is crucial [1]. The motion of a human walking up to the object and interacting with the object, i.e., grabbing, lifting, pushing, etc., is also important to characterize the typical ways humans interact with these objects. This research produces a library of data representing user biomechanics while interacting with those objects. The library will be used in future work to evaluate interaction with the objects and to design haptic manipulators that will mimic the objects in virtual reality. The haptic devices and virtual objects will then be used in user studies comparing virtual and real objects using the data from this library.

Our motivation for creating the library is somewhat unique. Many libraries, such as the KIT database [2], are used to create a database representing human–object interactions with multiple objects, but they usually focus on motion alone, and often strive to mimic that motion in related applications. Motion capture data from the KIT library, for example, is used to control robotic humanoids in a simulation [3] using a reference kinematics and a dynamics model of the human body called the master motor map (MMM). The KIT database is also used to segment bimanual manipulation from household tasks based upon human demonstration [4], which is also used to control humanoid robots. Other researchers have also used the KIT database to create motions for humanoid robots [5] based upon motions of humans.

While these research endeavors characterize human kinematics while interacting with an object, we also aim to characterize haptic interactions with objects. While the KIT database has been applied to create a taxonomy differentiating coordination patterns in human bimanual manipulation [6], our motivation is to eventually characterize human interactions with objects from both a biomechanics and haptics perspective, which will be used as a baseline for evaluating the haptic manipulators that we are developing. While the data from our library might be used to create a taxonomy like in [6], this is not a specific motivation for our research.

To characterize human kinematics and haptic interaction, our library comprises a combination of motion capture data, load cell data of the interaction between humans and different objects, and IMU (inertial measurement unit) readings of the object when it is being manipulated.

## 3. Related Work

### 3.1. Haptics

Haptics is the study of touch and motion. For us specifically, we are researching how subjects touch and move different objects from everyday life. The field of haptics is an ongoing research topic [7,8]. There are four main subareas in haptics: human haptics, machine haptics, computer haptics, and multimedia haptics [9]. Human haptics is more focused on physiological response, such skin stretching in response to forces, which is not related to this work. Machine haptics is more focused on design and control of a device to mimic haptic interactions, which will be more a focus in our future work designing and evaluating haptic manipulators based upon the library in this paper. In this study, we focus on the forces, torques, and manipulation of objects by humans and the way humans orient themselves to interact with these objects. Motion capture, load cells on the objects, and an IMU on the object track the human subject and provide haptic measurements when the subjects interact with the objects. Computer graphics haptics is more focused on the visual display of objects moving due to haptic interaction, which will be the focus of future work citing this library. Multimedia haptics is more focused on the ability of multimedia display to mimic haptic interaction, such as the work in [10], where environmental displays such as wind and mist were used to create haptic sensations, which is quite different from this work.

Adding load cells to objects that humans are interacting with has been performed before. Russo et al. studied humans balanced on a wooden beam while holding two canes with load cells in the base and motion capture to track the forces and human orientations used to balance themselves on the beam [11]. McClain et al. conducted a similar experiment of a human using a walker for load support, but used strain gauges on the tubes supporting the handles to measure interaction forces [12]. The data were used to design a new walker and its control systems to support similar forces. In this study, we use a 6-DoF load cell to obtain interaction forces, and evaluate how a user interacts with haptic manipulators mimicking a walker. We believe that the work presented here is unique, since the goal is to create a library of such biomechanical and haptic interactions with objects while standing and walking.

### 3.2. Similar Databases

There are many openly available human motion databases that include a wide range of information. These databases are essential for many different fields, such as computer vision, computer graphics, multimedia, robotics, human–computer interaction, and machine learning. Zhu et al. [13] provide an extensive survey of human motion databases, characterizing them by measurement techniques, types of motion and manipulation used to generate the data, and the types of data contained in the studies. Common data sources include marker-based motion capture, ground reaction force data, inertial measurements, video, and imagery. A wide variety of motions are studied, such as walking, lifting, kicking, dancing, and interaction with different objects. Some tasks require single-handed manipulation, and others require two-handed manipulation. Objects range from small household items, such as bottles, caps, and utensils, to hand-held tools, and large items, such as furniture, boxes, and doors.

Of the many databases described in [13], the work presented here is most similar to those focusing on manipulation of medium-to-large objects from daily life combined with locomotion, specifically where one- and two-handed manipulation is required. Human motion and kinematics, object motion, and kinetic interactions with the object are characterized. A brief review of the most related datasets is provided below and summarized in Table 1, which highlights the different types of data presented, the types of motion used to collect data, and the objects that are manipulated.

In [14], Bassani et al. provide a dataset characterizing human motion and muscular activity in manual material handling tasks, which is typical in the biomechanics and ergonomics fields. Users lifted and moved a small barbell weight with one arm and a medium-sized box using bimanual manipulation, amongst other tasks. Data were collected using an Xsens MVN suit (Movella, Henderson, NV, USA) composed of seventeen 9-axis IMU sensors and 8 sEMG sensor readings containing data from 14 subjects. Subjects stood in the N-pose in front of a table, lifted the object from the floor, kept it lifted with the elbow flexed at 90 degrees, and placed it on a table. Subjects then lifted the object from the table, carried it to another position, and placed it on the floor. Their box manipulation task is like the box manipulation task in our library, but our study uses the box as an instrument to measure interaction wrenches and uses motion capture to measure kinematics, which is unique, since our database provides a baseline for characterizing haptic interaction.

Geissinger et al. [15] used an XSens MVN with sparse IMUs to measure human kinematics during activities of daily life on a university campus, driving, going to a variety of different stores, working in a machine shop and laboratory, exercising, and at home. This generated a large dataset across 13 subjects, which was then used to train deep learning networks to predict upper body motion and full body motion. While some of the activities, such as walking up to a door and opening it, pushing a cart, or lifting a box, are like those in our study, they only focus on the kinematics of the human, whereas the database presented here also characterizes kinetic interaction with the object and its resulting motion.

The hand–body–object dataset (HBOD) includes data of subjects moving and interacting with a screwdriver, hammer, spanner, electrical drill, and rectangular workpiece with one hand [16]. Data are derived from 12 IMUs placed on body segments and a Cyberglove 2, which together measure body and hand kinematics. In this case, motion capture markers were used to measure the posture of the objects being manipulated. Five subjects then lifted and placed objects at high positions and performed tool manipulation. While the measurement of human and object kinematics are similar to our database, ref. [16] focuses more on dexterous kinematics, and kinetics are not measured. Likewise, the objects being manipulated are quite different than those presented in this paper.

The industry-oriented dataset for collaborative robotics includes data of human postures and actions that are common in industry [17]. Like HBOD, [17] measures whole body kinematics using both IMUs and a glove with flexion sensors to measure hand kinematics. Similarly to our work, they also use marker-based motion capture to measure human kinematics. Their gloves use pressure sensors to detect contact with the objects, but this does not provide kinetic information about interaction forces as we do in this paper. The study emphasizes tasks typical of assembly lines, which are mainly focused on picking up small parts, carrying them, and then assembling them with other objects. They do, however, evaluate lifting larger loads and moving them to a shelf, which is like the box lift task in our study. Unlike HBOD and this paper, the object motions in [17] are not measured. Measurements from 13 subjects are reported in [17], in which two cameras are used for video recording tasks, but data are not derived from them.

The BEHAVE dataset [18] includes a large set of images of eight subjects interacting with objects in natural environments. This dataset contains multiview RGBD frames with accurate pseudo-ground-truth SMPL (skinned multi-person linear model). The objects manipulated include various boxes, chairs, and tables, as well as a crate, backpack, trashcan, monitor, keyboard, suitcase, basketball, exercise ball, yoga mat, stool, and toolbox. Interactions include lifting, carrying, sitting, pushing, and pulling, as well as unstructured free movement combined with object interactions. One- and two-handed manipulations are provided with hands, as well as manipulations using feet. Imagery data does not measure kinematics and kinetic interaction directly, but the authors do provide computer vision code to demonstrate a basic reconstruction of kinematics using the data. It is not clear if such information is sufficient for characterizing haptic interaction.

The KIT database includes a dataset of full-body human and object motion capture using one- and two-hand manipulation [2]. This database has 43 different subjects and 41 different objects with raw data and post-processed motions. This dataset includes a full body motion capture marker setup with 56 markers and markers placed on each of the objects to analyze object movement. The objects and interactions include tasks such as opening a door, manipulating a box, walking up and down stairs, and interaction between subjects. This is a very extensive database and includes a plethora of other objects, but it is mainly focused on kinematics. Kinetic interaction is not measured.

While these studies have provided detailed insight into how people interact with specific objects, our end goal focuses on haptic interaction with a series of everyday objects combined with locomotion. The database described in this paper includes full body motion capture and object motion capture data, which is only present in a few studies, such as [2]. Unique to our database is object instrumentation using IMU measurements of the object’s motion when a human is moving the object and measurements of interaction wrenches using 6-DOF load cells. This allows the detailed measurement of forces and torques exerted on the object combined with the object’s accelerations and position, which should be sufficient to characterize haptic interaction with the object. This new information can be used to characterize the different object models for future applications, such as haptics and virtual reality (VR). Further unique to this study is that this haptic interaction is combined with measuring human motion during that interaction, which allows for the simultaneous characterization of human gait and kinematics during haptic interaction. This knowledge will be helpful for validating interactions with future haptic devices mimicking these objects in a virtual world.

## 4. Tasks

**Figure 1:**
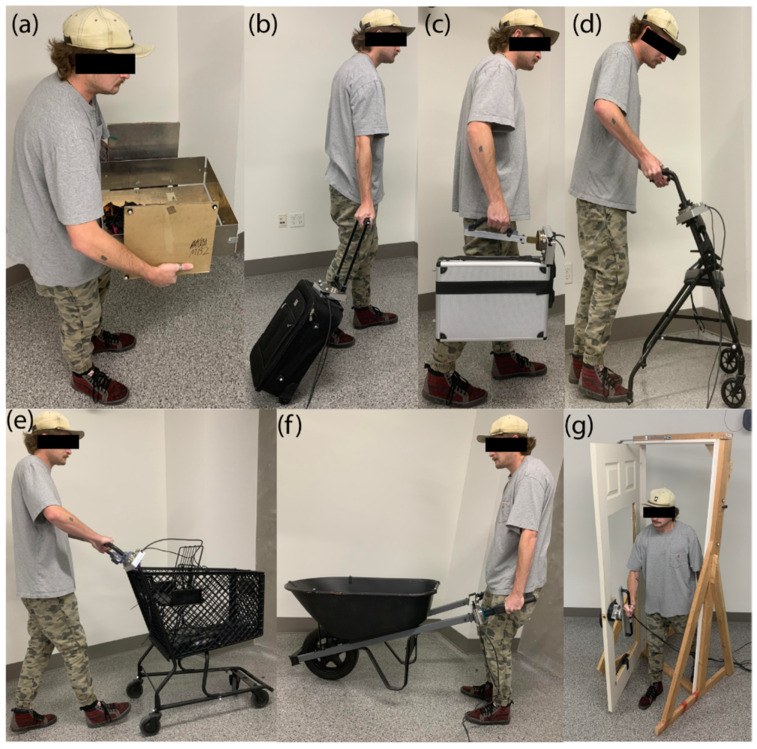
Tasks performed in everyday life: (**a**) manipulating a box, (**b**) walking with luggage, (**c**) carrying a briefcase, (**d**) using a walker, (**e**) pushing a cart, (**f**) manipulating a wheelbarrow, and (**g**) opening a door.

Several activities of daily life have been identified as motivating tasks for this research based on coordination of locomotion and manipulation. The first task uses two-handed oppositional grasp and is shown in Figure 1a, where a user is grasping and lifting a box on a virtual table. The next task uses a piece of luggage, also known as a roller-board, where the user supports the luggage by the handle and pulls it behind them as they walk, Figure 1b. This task is proposed as an example of a person walking through an airport. A task using a briefcase, Figure 1c, is shown in the user’s right hand, which is carried like a person walking to work. A walker usage task is presented, Figure 1d, which is motivated by our prior work with subjects with Parkinson’s disease [19] and spinal cord injury [20] where the walker is used to help support the user while they walk. Walker usage usually requires the user to lift the handles slightly and push it to locomote. The shopping cart task, shown in Figure 1e, requires the user to push the cart using different levels of force in each hand to control the cart direction. Figure 1f illustrates a task where the user operates a wheelbarrow, which is common for yard work around the home. This requires the user to lift the back of the wheelbarrow with the handles and then push it forward while walking. They may slightly tip side to side to control steering. Depending on the load in the wheelbarrow (e.g., dirt or concrete versus wood chips or leaves), this can be incredibly demanding, requiring both strength, coordination, and balance. Hence, we will use wheelbarrow operation to display more physically challenging tasks to the user. The door opening task is proposed, as shown in Figure 1g, which was selected since this is consistently repeated in everyday life. The door is configured to open inward and pivot on its right side, such that the user pushes the door open with their right hand and steps through.

## 5. Methods and Procedures

This paper presents methods and procedures used to create a database describing how humans interact with the objects which were presented in Section 4. The human walked up to these objects, grabbed the handles, lifted/lowered, pushed, or walked with a steady gait while grasping/manipulating the object. During the study, information such as forces, torques, object motion, human kinematics, and human gait were collected. Furthermore, 6-DOF load cells were incorporated into the object’s interaction areas, such as handles, record forces, and torques applied to the object by the human. Body and object kinematics and dynamics were studied using full body motion capture and IMU readings of the object motion when a human interacts with the object.

### 5.1. Motion Capture Volume

The motion capture system consisted of a 10-camera VICON motion capture system with a mixture of MXF20 and MXF40 cameras (Oxford, UK). The capture volume was designed to capture full body motion in an 8.2 by 4.5 by 2 m sized space. This ensured that we captured full body data for a subject who is 1.9 m tall, locomoting for multiple gait cycles and interacting with the objects naturally. The room was sufficiently large so that users could begin walking and establish a gait pattern.

### 5.2. Marker Set

**Figure 2:**
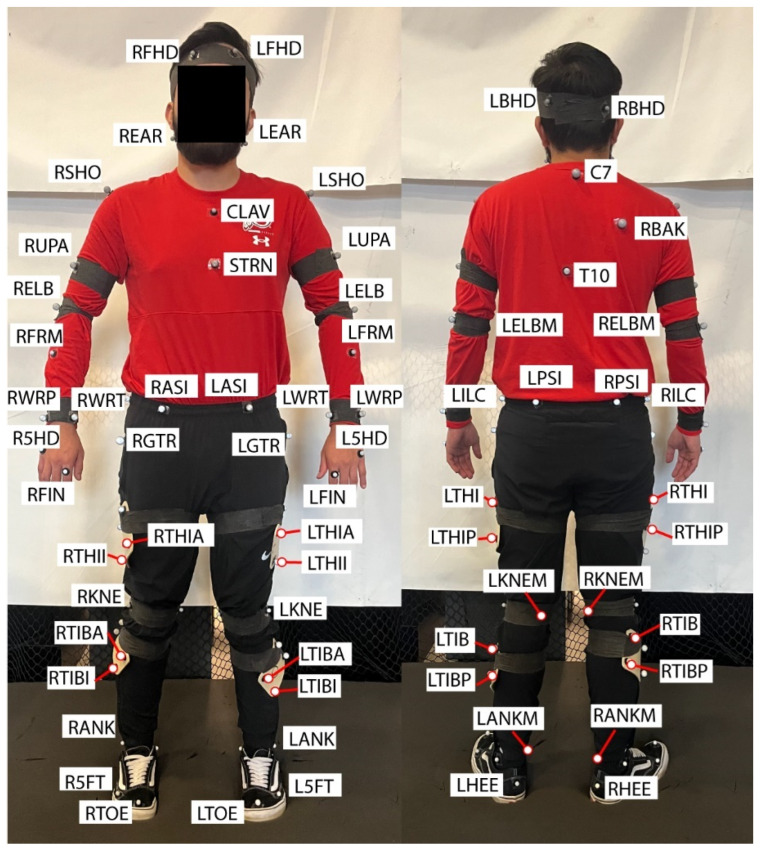
Motion capture marker setup [21,22].

The full body marker setup is shown in Figure 2. Reflective markers were placed on all main segments and on rotational joints [21,22]. A minimum of three markers were placed on each of the segments to fully define the segment and record its movement. The motion capture setup was found to be sufficient enough to obtain full body motions for all sizes of participants in the study. These data were exported in CSV and C3D files to be viewed in the database.

### 5.3. Instrumentation and Syncronization

**Figure 3:**
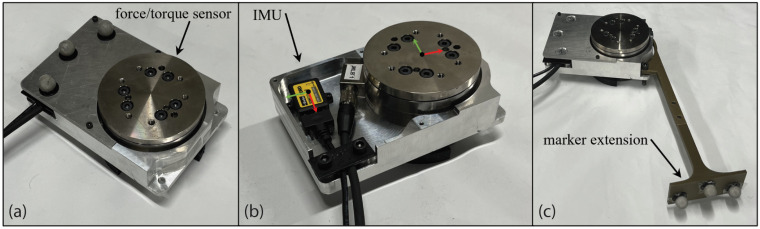
The load cell and IMU case (**a**) with the cover on, (**b**) with the cover off, (**c**) with the marker extension.

Each object was instrumented with force/torque sensors, an IMU, and motion capture markers. The force/torque sensors were 6-DOF sensors from ATI Industrial Automation (9105-TIF-OMEGA85), (Apex, NC, USA). The IMU was a Parker Lord Microstrain IMU, (3DMGX5-AR), (Williston, VT, USA) which can capture a wide variety of different readings. We are mainly interested in the acceleration of the object when it is manipulated by the user. One of the load cells and the IMU were encased inside the IMU/load cell casing to keep the IMU safe and have the respective coordinate frames stay consistent between the load cell and the IMU (Figure 3). The load cell/IMU casing had a marker extension with VICON markers on it designed to produce an unobstructed coordinate frame when connected to the different objects. The second load cell case was similar, but did not have an IMU inside of it.

Loads were applied to the objects via handles that were attached to the force/torque sensor, as indicated in Figure 3a–c, which is the output of the load cell. As a result, most of the mass of the load cell and the case supporting it were included in the mass of the object being manipulated, as indicated in the next section. Since the handles were attached to the output of the load cell, the load cell could not measure the forces/torques due to the handle’s motion, but they were small compared to the forces/torques required to manipulate the objects. For example, as indicated in the next section, the handles and output plate had a mass of 0.24 kg to 1.2 kg, whereas the masses of the objects were ~8 kg to ~40 kg. The case, IMU, and load cell had a mass of 1.5 kg and measured 15.24 by 10.16 by 4.62 cm.

Each sensor was logged on a separate computer, which required a method of data synchronization. The dSpace system sent a chain of impulses to the VICON DAQ (Oxford, UK) to line up the data between the load cell and the motion capture data. To synchronize all of these data, the object was given 10 short impulses by the subject, resulting in accelerations in the IMU, and force measurement in the force/torque sensor. Displacements in the motion capture data, which are synchronized in time, can be used to check the synchronization between all the different datasets. Synchronization between the different sensors/computers is essential to keeping the data consistent.

### 5.4. Object Instrumentation

#### 5.4.1. Box

**Figure 4:**
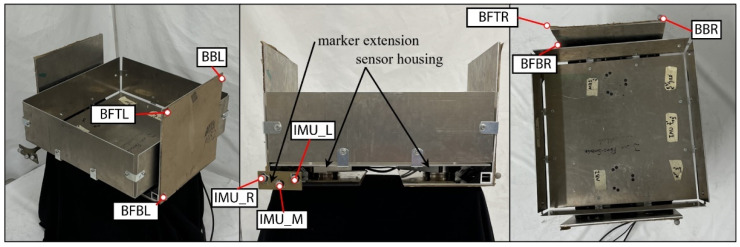
The box apparatus.

The box used in this study was heavy and large, so this was more challenging than other tasks. The box object was constructed to model a 50.5 × 45.7 × 45.7 cm cardboard box with moderate rigidity. The box weighed 8 kg and had an additional 1.4 kg weight placed inside. Figure 4 shows the box object, which comprised three parts: the main body of the box, and then two separate sides. The main body was constructed of thin, aluminum plates with small brackets for added rigidity. The sides were made of large aluminum brackets with cardboard mounted externally, allowing the user to feel the texture of cardboard during manipulation. The two force/torque sensors were mounted between the main body and each of the sides. The IMU was encased inside the IMU/load cell casing, and was between the main body and the left side, as shown in Figure 4.

#### 5.4.2. Luggage

**Figure 5:**
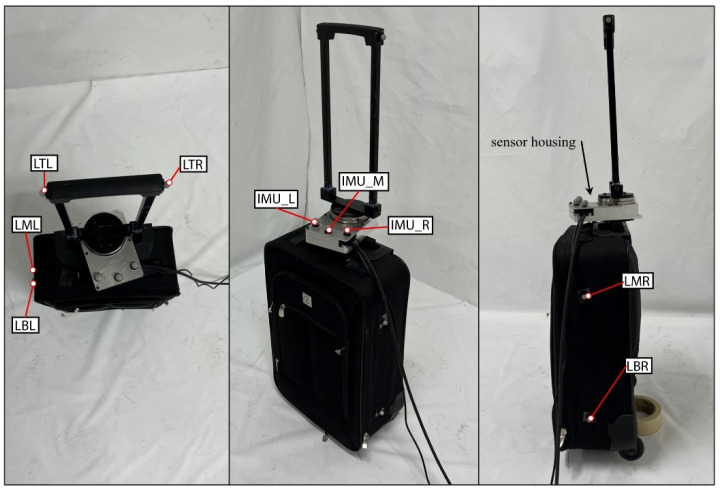
The luggage/roller board apparatus.

The luggage object was a 48.3 by 33 by 17.8 cm roller luggage compartment with an instrumented handle, as shown in Figure 5. There was a 4.5 kg weight added inside the luggage compartment to simulate a packed piece of luggage. The luggage itself weighed about 2.3 kg. The sensor housing containing the load cell and IMU was between the luggage compartment and the handle. It was positioned here to measure the forces exerted by the human when holding the handle and walking with the object. Motion capture markers surrounded the compartment and were on either side of the handle. There were also markers placed on the sensor housing to know the location of the load cell and IMU.

#### 5.4.3. Briefcase

**Figure 6:**
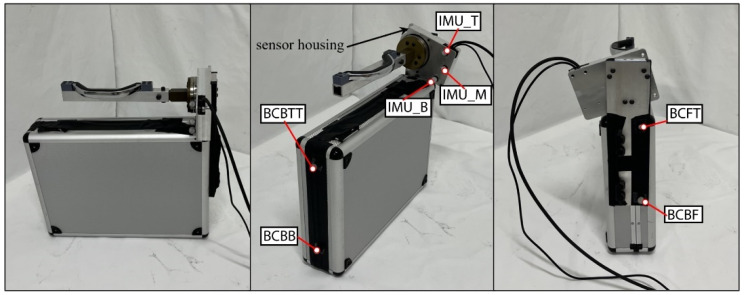
The briefcase apparatus.

The briefcase was a 48.3 by 12.7 by 45.7 cm briefcase with an articulating handle, as shown in Figure 6. The briefcase had weight added inside of it to simulate a full briefcase weighing 8 kg. The briefcase handle was prepared by detaching it from the briefcase and putting it on a fixture consisting of aluminum and 3D-printed parts. This fixture was connected to the load cell, which was connected to the sensor which also housed the IMU. The handle was able to rotate exactly the same as the original briefcase design. An aluminum plate was bolted to the side of the hard case of the briefcase to be able to mount the handle and the sensor housing. The handle was in the relative location, as it was when connected to the briefcase, but elevated slightly to accommodate the sensor housing. Motion capture markers were placed around the briefcase and on the sensor housing.

#### 5.4.4. Walker

**Figure 7:**
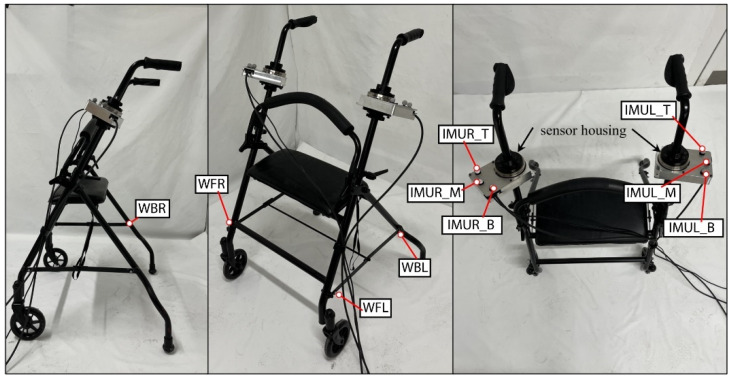
The walker apparatus.

The walker object was a modified assistive walker, with the handle height being 94 cm high and the handles being 61 cm apart with a weight of about 7.5 kg, as shown in Figure 7. The load cells/IMU casings were located between the handles and the adjustable height bars. The height was adjustable, but set to a position that an average person can comfortably use. Motion capture markers were placed around the lower frame of the walker and the load cell/IMU casings.

#### 5.4.5. Shopping Cart

**Figure 8:**
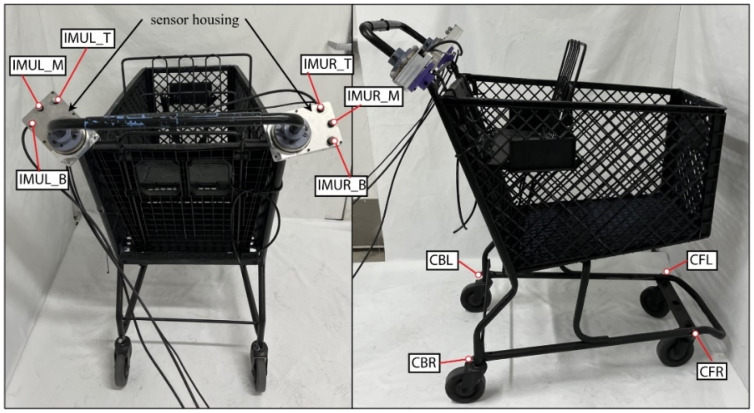
Shopping cart apparatus.

The shopping cart was a standard shopping cart typical of a US grocery store, as shown in Figure 8. The shopping cart was 91.5 by 61 by 112 cm, with the handles located 112 cm above the ground. The shopping cart weight was 19.5 kg, and there was also a 22.7 kg plate fixed inside the shopping cart to simulate the cart carrying groceries. The cart was painted matte black so it did not affect the motion capture cameras when capturing datasets. The load cells and IMU were located between the handles, which were split, and the shopping cart body. The frame of the handles was cut so that they sat at the same location as a normal shopping cart. There were motion capture markers placed around the lower frame, near the wheels, and around the top of the cart basket.

#### 5.4.6. Wheelbarrow

**Figure 9:**
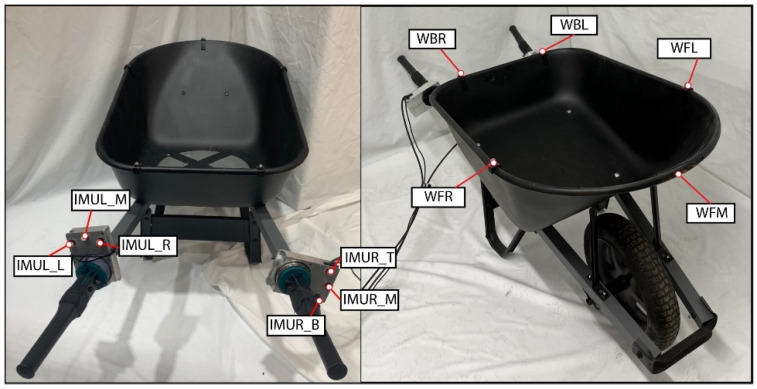
Wheelbarrow apparatus.

The wheelbarrow was a standard steel wheelbarrow purchased from Home Depot, as shown in Figure 9. The wheelbarrow was a 68.58 by 67.8 by 115 cm fixed wheelbarrow which weighed 16.3 kg. The wheelbarrow also had a 22.7 kg plate in it to simulate the wheelbarrow carrying a load, such as dirt. The load cell and IMU cases were located between the chassis of the wheelbarrow and the handles. The end of the handles were in the same position as they were originally, so as to not change the overall design. There were motion capture markers located around the bucket of the wheelbarrow and on the load cell/IMU casings. The wheelbarrow was painted matte black to prevent interference with the motion capture system.

#### 5.4.7. Door

**Figure 10:**
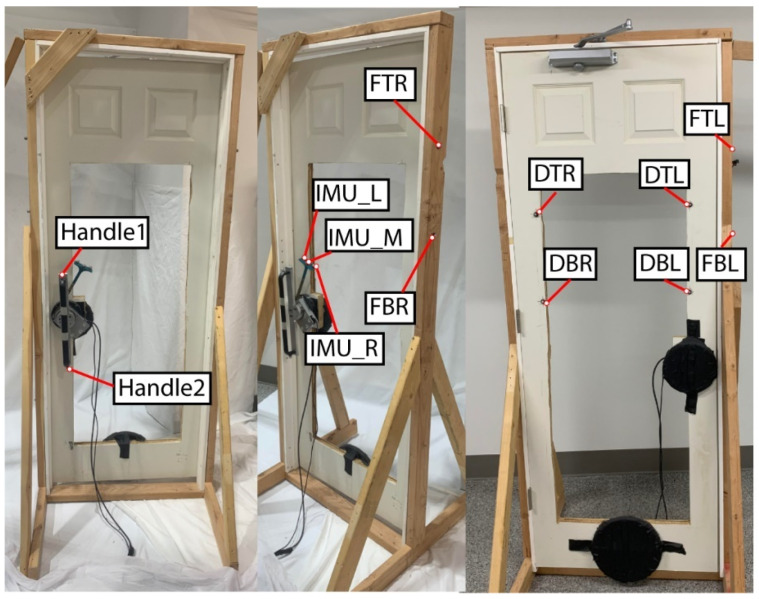
The door apparatus.

The door was a standard solid core-molded composite interior door, purchased from Home Depot (Item No. THDQC225400566). The door was 76.2 cm wide by 203.2 cm high, and weighed 25.4 kg. The door was cut out in the middle to allow the motion capture cameras to see through the door and view the motion capture markers on the front of the subject (Figure 10). Due to cutting out a large portion of the door, added weights were placed on the door to simulate a heavy door weighing 15.87 kg. The door had a door closer, so there was added tension on the door, and the door closed by itself. The door handle was a standard long door handle, which was affixed to the load cell and IMU case, and then attached to the door where a standard door handle would be. The load cell and IMU case had the extension on it to put the motion capture markers in an area where they can be seen by the motion capture cameras. There were also motion capture markers placed on both sides of the door and around the frame of the door so the door angle could be found when opening it.

### 5.5. Transformations

**Figure 11:**
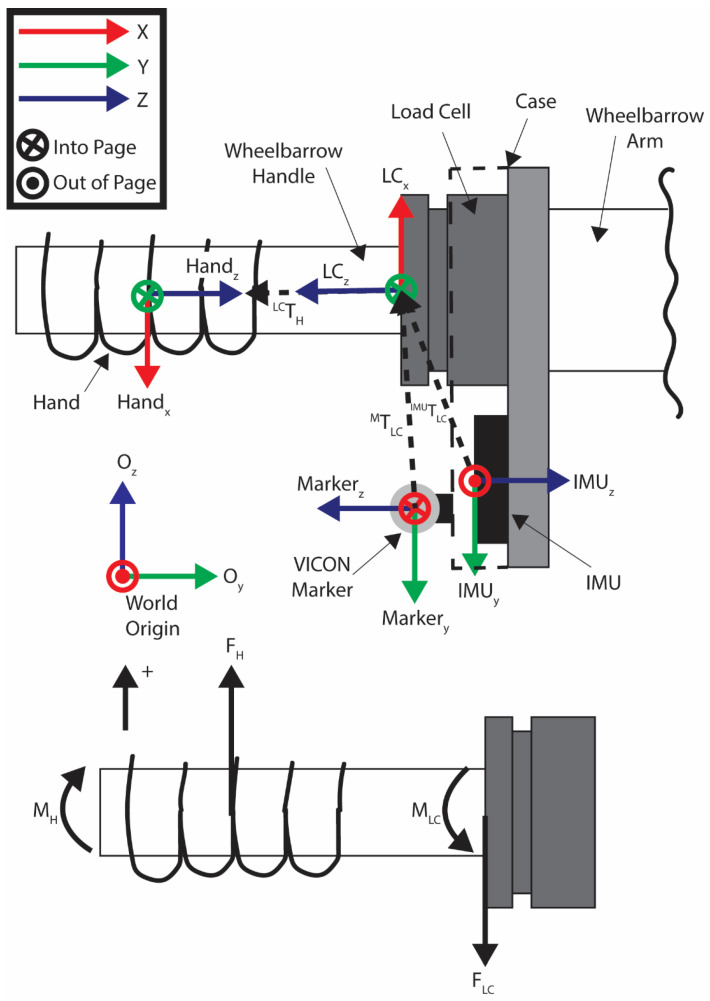
Diagram of the wheelbarrow handle, load cell, and IMU case with VICON markers, and the wheelbarrow arm. The top diagram shows the coordinate frames of the VICON markers relative to the motion capture origin, the marker, IMU, load cell, and the subject’s hand. The transformations, T, are shown between the marker and load cell, IMU and load cell, and the load cell and subject’s hand. The coordinate frames (blue, red, and green vectors correlating to x, y, and z, respectively) show the orientations of each of the sensors relative to the world coordinate frame. The lower image shows the forces at the hand and how they relate to the load cell forces.

The information provided in the library from the sensors (IMU and load cell) was in the respected coordinate frame of the sensor. To move that information from the sensor to the handle where the interaction took place, the information needed to be manipulated through a transform to calculate the forces and torques at the handle. This provided the haptic information between the subject and the handle of the object. The calculation was performed by taking the sum of forces and moments at the load cell. The top diagram in Figure 11 shows a wheelbarrow handle diagram of the world origin, IMU, VICON marker, load cell, and subject hands coordinate frames with the transformation between them. The lower image in Figure 11 shows the forces and moments acting on the wheelbarrow handle.

An example of how to perform this calculation is provided. Consider that $F_{H}$ is the force vector at the handle exerted by the subject’s hand and $F_{LC}$ is the force vector measured at the load cell. Summing the forces provides,

(1) $$\sum F=F_{H}-F_{LC}=0$$

Which can be solved to show that the load cell forces are equal and opposite to the handle forces, such that $F_{H}=F_{LC}$. Summing moments about the load cell then results in,

(2) $$\sum M={-M}_{H}+M_{LC}-F_{H}\times T=0$$

where $M_{H}$ is the moment exerted on the handle by the subject, and $M_{LC}$ is moment acting on the load cell. *T* is the homogeneous transformation matrix from the load cell to the handle of the object

(3) $$T=\left[\begin{array}{cc} R & d\\ 0 & 1 \end{array}\right]$$

which is constructed from the rotation matrix, R,

(4) $$R=\left[\begin{array}{ccc} r_{11} & r_{12} & r_{13}\\ r_{21} & r_{22} & r_{23}\\ r_{31} & r_{32} & r_{33} \end{array}\right]$$

from the load cell to the handle, and d is the displacement from the load cell to the handle,

$$d=\left[\begin{array}{c} d_{1}\\ d_{2}\\ d_{3} \end{array}\right]$$

The moment equation can then be solved to calculate the moment at the handle, $N_{H}$, as follows:

(5) $$M_{H}=M_{LC}-F_{LC}\;\times \;T$$

Combined, $F_{H}$ and $M_{H}$ indicate the forces and moments supported by the user.

The transformation, T, for each object handle is provided in the data library, which allows load cell data to be transformed to calculate forces and moments exerted by the user for each object. Transformations between the marker to the load cell, IMU to the load cell, and the transformation between the load cells are also included in the data library. This can be used to calculate the position, velocity, and acceleration of the object being manipulated using standard coordinate transformation techniques [23].

### 5.6. Object Interaction Protocols

The database included a variety of human motion capture files, object motion capture files, and sensor files specific to interactions with the objects mentioned in Section 3. Separate protocols for each object were used to provide similar interactions amongst the subjects. While all interactions involved walking and manipulation of the object, they were all slightly different due to the unique ways humans interacted with each of them. We generally evaluated multiple interactions with each object, such that lifting an object was considered separately from walking with the object. This allowed us to decouple the two types of interactions for evaluation in future studies. Likewise, tasks involving walking while manipulating the object initiated the gait with the left foot, allowing a common gait pattern to be established in all the trials. All interactions were designed to mimic natural interactions with the objects, although interactions were segmented, as indicated above.

(1) Box: The box had two types of interactions. The first was lifting. The box was sitting on a stand. The subject started standing straight up, then bent down and grabbed the sides of the box with an oppositional two-handed grasp and then lifted the box. They then lowered the box back down to the stand, let go, and stood back up. In the second interaction, the subject carried the box while walking. The subject started at one end of the capture volume holding the box with a two-handed oppositional grasp, and then walked through the volume. They started with their left foot and walked to the other side of the volume with a steady gait while holding the box.
(2) Luggage: The subject started at one end of the capture value holding the handle of the luggage. They then walked through the capture volume, pulling the roller luggage behind them. They began walking with their left foot first, while keeping a steady gait until they reached the other side of the capture volume.
(3) Briefcase: The briefcase had two different types of interactions. The first was lifting. At the start, the subject was standing straight up, then bent down and grabbed the handle, and then lifted the briefcase. They then lowered the briefcase to the ground, let go, and stood back up. In the second interaction, the subject carried the briefcase while walking. They started at one end of the capture volume while holding the briefcase in their right hand. They then walked, starting with their left foot, through the volume to the other side, with a steady gait, while holding the briefcase.
(4) Walker: In this interaction, the subject walked through the motion capture volume using the walker for support. They started at one end of the volume with their hands grasping the walker handles. The subject then lifted the handles slightly and pushed the roller walker forward while extending their arms. They set the handles down and then applied downward force on the handles to support their weight while stepping towards the walker. The process was repeated until the subject traversed the capture volume.
(5) Shopping cart: The subject approached the shopping cart and then pushed it through the motion capture volume. The subject started in a standing position a step back from the shopping cart. They stepped towards the cart and placed their hands on its handles; both of their arms were lifted up and grasped the shopping cart. They then pushed the shopping cart through the volume while walking.
(6) Wheelbarrow: The wheelbarrow had two types of interactions. The first was lifting the handles. The subject started in a standing position between the handles. They then bent down, grabbed the handles, and lifted the wheelbarrow handles. The handles were then lowered until the back of the wheelbarrow again contacted the ground. The subject then released the handles and stood back up. In the second task, the subject pushed the wheelbarrow through the volume while walking. The subject started at one end of the capture volume while holding the wheelbarrow handles, meaning that the back of the wheelbarrow was lifted and the front was balanced on its wheel. They then walked through the volume while pushing the wheelbarrow. They started walking with their left foot, developed a steady gait, and pushed the wheelbarrow through the capture volume.
(7) Door: The subject walked up to the door, opened it, stepped through, and released the door handle. They began standing a few steps away from the door. Unlike the other walking trials, the subject first took a step with their right foot due to the location of the door so they could more naturally open the door and step through it. After the right step, they stepped with their left foot towards the door while also reaching with their right hand to grasp the door handle. They then pushed the door open with their right hand and walked through, with their left foot leading. This is due to the fact that it was a right-handed door, meaning that the hinge was on the right side of the door and the handle and opening were on the left side. They then finished stepping through the door with their right foot and released the handle, allowing it to start closing. They then took a half step with their left foot, such that it evened up with their right foot. The subject was then standing straight up, and the door had closed behind them.
(8) Gait: Natural gait was also measured, since so many of the tasks involved walking with objects. This allowed a baseline comparison in future studies so that the effect of handling the objects could be considered. The subject stood at one end of the capture volume and started walking with their left foot. They walked across the capture volume and their gait was recorded.

## 6. Data Processing

### 6.1. VICON

Processing started with the data collected via our VICON motion capture system. Marker registration was verified first to ensure that all markers were continuously registered through the motion sequence, since markers may be lost occasionally. VICON Nexus data gap-filling (i.e., spline, cyclic, and pattern filling) was applied to resolve unregistered markers. For small gaps, a mixture of cyclic and spline gap-filling was applied. Cyclic filling used information from the whole trial. Since we were repeating gaits, cyclic was a great option due to the repetitive nature of the study. Spline filling used cubic spline interpolation of the points around the marker gap. Pattern filling was used for large gaps where cyclic and spline filling failed. Pattern filling referenced other markers that experience the same trajectory characteristics (rigid bodies) on the same segment.

After marker filling, VICON data were then exported. Exporting was the same for all studies, no matter the object or trial. For each full trial, trajectory data were exported in CSV data files and C3D motion files while the trigger data were exported in CSV files. Trigger data were generated by the dSpace system, as indicated later in this section. Trajectory data included motion capture data from both the subject and object.

Full trial data were also segmented in Nexus to create short segments for repeated tasks. For example, a subject may have lifted an object multiple times in a full trial, or may have pushed an object multiple times in a full trial. The segmented trajectory data were trimmed to windows where only the whole human and object marker sets were visible. Segmented trajectory data are provided in CSV data file and C3D motion file formats in the database.

### 6.2. AddBiomechanics

AddBiomechanics is an open-source motion capture pipeline developed at Stanford University that uses optimization to process uploaded motion capture data faster and more efficiently [24]. The C3D files from the previous subsection were uploaded to AddBiomechanics along with subject demographic, height, and weight information. The C3D data were combined with a musculoskeletal model of the subject. The OpenSim musculoskeletal model by Rajagopal et al. [25] was adapted to use our marker set, which was included in the library. The single markers used by Rajagopal at the thigh and shank were replaced by the marker rigid marker plates that we used at these locations. The four markers used by Rajagopal at the shoulder were replaced by our single marker. Our marker set also included an additional marker on the hand and three extra markers on the trunk, which were added to the model. The modified musculoskeletal model is provided in the database and is stored in AddBiomechanics with the data.

AddBiomechanics was then used to process the data. AddBiomechanics first estimated the functional joint centers and then created an initial guess for body segment scales and marker registrations [24]. Bilevel optimization was applied to fit the model geometry and kinematics to the marker data. AddBiomechanics was then used to produce exportable files that include joint and segment rotational angles. The files produced byAddBiomechanics were marker error (CSV), the joint and segment angles (MOT), and PDF previews of the data. All of these data are available in AddBiomechanics and in our database.

### 6.3. dSpace/Load Cells

The dSpace file was presented as a MAT file type, and contained the information from both of the load cells (force and torque). This file included time syncing data (i.e., trigger data) and force and torque measurements. Both raw and filtered data were included. Filtered data were also included by using a low pass filter with a 20 Hz cutoff frequency.

### 6.4. IMU

The IMU data were presented as a CSV file, and included data reported by the IMU located on the object. Data were transmitted by serial communications to the dSpace computer using the SensorConnect 15.6.4 software. The information in this file included the acceleration readings of the object when it was interacted with, and the gyroscopic readings when the object was manipulated.

### 6.5. Final Data Synchronization and Export

**Figure 12:**
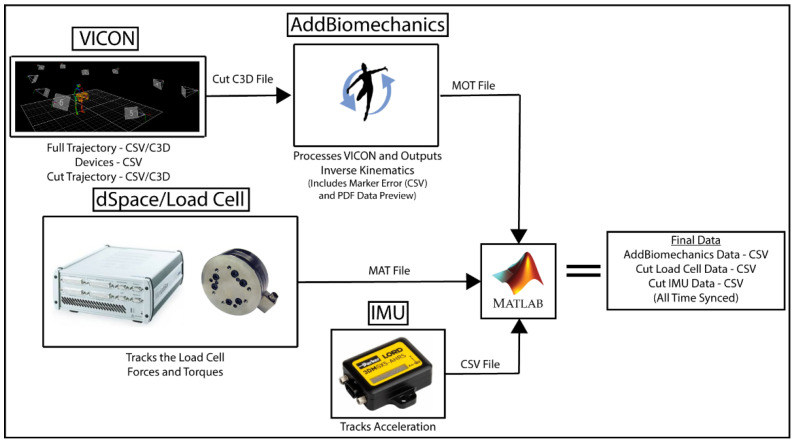
Data processing flowchart.

Once the data processing, described in the previous subsections, was complete, final synchronization and file generation was performed, as shown in Figure 12. Data were synchronized and exported for each subject, object, and interaction separately. A MATLAB 2024b script first loaded the data:

- VICON trajectory CSV,
- VICON trigger data CSV,
- Cut VICON trajectory CSV,
- AddBiomechanics MOT motion file,
- Load cell MAT file,
- IMU CSV file.

This MATLAB script then synchronized the different datasets. The VICON trigger data CSV contained analog measurements of the trigger signal which was produced by dSpace when the measurements started. The same trigger signal was recorded in the load cell MAT file, and was used to align the timestamps of the two datasets. The load cell MAT file and IMU CSV file timestamps were then synchronized by detecting the 10 taps, as indicated in Section 5.3. The cut VICON trajectory CSV timestamps were the same as those in the VICON trajectory CSV, so the timestamps in the Cut VICON data are then used segment the Load Cell and IMU data correspondingly for each trial. This allows the cut data to represent both the kinematics and kinetics for each trial separately. AddBiomechanics shifts the start time for each trial to zero to represent the start of each trial uniformly. The segmented Load Cell data and IMU data for each trial are likewise shifted to zero to correlate to the AddBiomechanics data. MATLAB exports the synchronized Final Data (AddBiomechanics, Load Cell, and IMU) in CSV format.

## 7. Results

### 7.1. Subjects

The dataset currently contains data from six subjects, which will be expanded as this project continues. The subjects are all healthy males ranging from 18 to 30 years old. They range from 1.6 to 1.9 m in height and 56.6 to 133 kg in weight. Data were collected according to University of Utah IRB #00123921.

### 7.2. Datasets

Our dataset includes a few different datasets from the different stages of processing, as shown in Figure 12.

**Figure 13:**
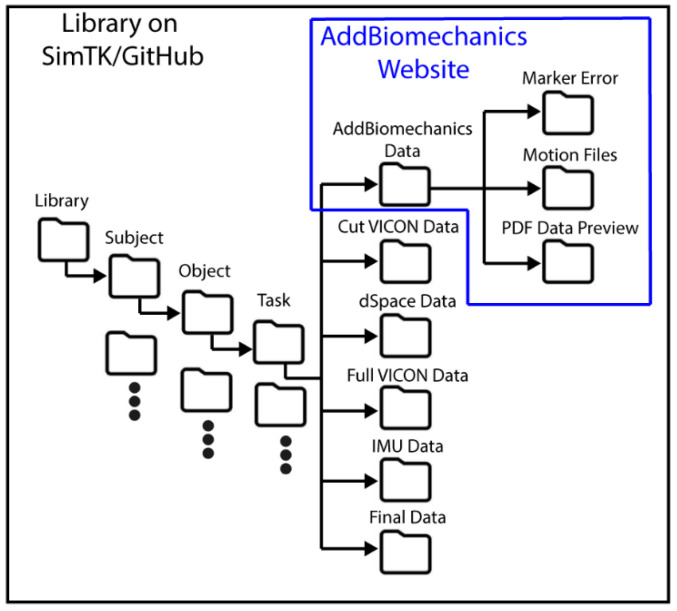
Database file organization.

(1) The main dataset is located on GitHub and SimTK and contains all the files described in Section 6 and shown in Figure 13. Data are arranged by subject, object, and task. The AddBiomechanics folder contains the data generated from the AddBiomechanics which is the marker error (CSV), motion files (MOT), and PDFs that show a preview of the data in the motion files. The cut VICON data folder contains the windowed marker data from VICON (CSV/C3D). The dSpace data contains an MAT file of each load cell and additional data needed for time syncing. The full VICON data folder contains the whole marker trajectory file for each task (CSV/C3D) and the trigger data file for syncing (CSV). The IMU data folder contains the IMU sensor data (CSV). The final data folder contains the synced AddBiomechanics full body joint and segment angles (CSV), the force and torque from the load cells (CSV), and the IMU data (CSV) of the windows of the VICON data. The entire library of data is available on GitHub and SimTK.
(2) The AddBiomechanics data are also available on the AddBiomechanics website and contain everything that is processed through AddBiomechanics, as shown in Figure 13. It also contains the uploaded files used to generate the AddBiomechanics data. It contains the PDF, the marker errors, and the motion files described above. It contains the marker data in a (TRC) file and the OpenSim models used to obtain this information. AddBiomechanics is provided so that other researchers can evaluate the data processing, and have ready access to raw and processed data.
(3) The main dataset is also available on GitHub and SimTK, and contains everything that is in the main database file described above. There is also the OpenSim model used for AddBiomechanics (OSIM), example processing code (MATLAB), and the homogeneous transformations (CSV) described in Section 5.5, which are available in the library.

### 7.3. Data Sample

**Figure 14:**
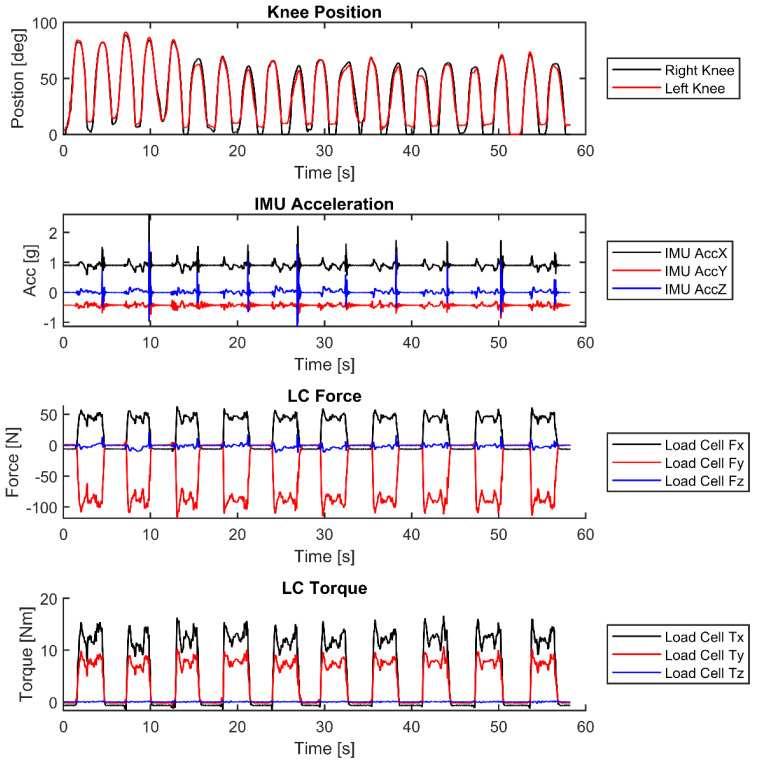
Example of typical data showing knee rotation from AddBiomechanics, IMU acceleration of the object when it is being manipulated, and force and torque from the load cell of the subject lifting and manipulating a briefcase.

There is a large amount of data in the library, and many different actions/objects. An example of the data is shown in Figure 14. Using the cut dataset from VICON, the AddBiomechanics dataset was generated. An example of the motion data included in the AddBiomechanics output is shown in Figure 14. The data shown are the right and left knee angles of a subject picking up a briefcase and then setting it back down ten times. These are only a subset of the kinematic angles available in the dataset. The dataset contains joint angles and segment angles of the whole body in the MOT files in the AddBiomechanics folder. As seen from the knee data, the angles of both knees increase as the subject bends down and reaches a position to grab the briefcase, and then it decreases back to ~zero in a standing position. The subject then bends back down to place the briefcase on the ground and releases the handle.

Example data from the load cell and the IMU are also presented in Figure 14. These data are aligned with respect to the time of the cut VICON and AddBiomechanics data. When the object is interacted with, the load cell and IMU signals vary. An example of the accelerometer data is shown in Figure 14. Spikes correlate to when the briefcase is picked up and when it is set back down. Non-zero forces and torques between the spikes correlate to the load cell data measured while the subject lifts and lowers the briefcase. The load cell data then returns to ~zero when the briefcase is set on the ground and released.

### 7.4. Data Analysis Example: Wheelbarrow

Data from the library are intended to be used to characterize how subjects interact with large objects using one- and two-handed manipulation while locomoting. Such information is beneficial to the biomechanics and robotics communities, where gait, interaction forces, and object motion can be studied. The biomechanics community, for example, may be interested in gait changes between normal walking and walking while carrying an object. Data are commonly analyzed to provide data such as spatiotemporal gait characteristics (e.g., stride length or cadence, etc.) [19,26,27], biomechanical characteristics (e.g., knee range of motion), joint trajectories (e.g., knee joint angle vs. gait cycle) [26,28] and cyclograms (e.g., comparing joint angles, such as knee vs. hip angle for one leg [28,29] or left vs. right angles for the knee joint [27]). Data from the library can provide such information, but require additional data processing, as described below. In the first example, we characterize a user lifting the wheelbarrow. In the second example, we characterize a person walking with and without the wheelbarrow.

#### 7.4.1. Time Series Data: Wheelbarrow Lift

Data can be analyzed to characterize typical results during an interaction. In this case, we show how data from lifting and lowering the wheelbarrow repeatedly can be processed to identify mean values and confidence intervals during the lifting motion. These data are cut using the AddBiomechanics right knee data. In this case, we show how the data can be analyzed to determine when a user is lifting the wheelbarrow. Data cutting analyzes 25 knee angle points moving along the signal. As the user crouches down to reach the handle, the knee angle increases, which produces positive slope between measurements. The user will eventually stand up with the wheelbarrow handle, which causes the knee angle to decrease. The transition from crouching to lifting is determined by when a positive slope has been detected 24 times and then a negative slope occurs at the next point. As the user lifts the wheelbarrow, knee angle measurements continue to decrease, producing negative slopes. When a negative slope has been detected 24 times and the knee angle starts to increase, a positive slope is determined, which indicates that the user is beginning to crouch down with the wheelbarrow, marking the end of the lifting.

**Figure 15:**
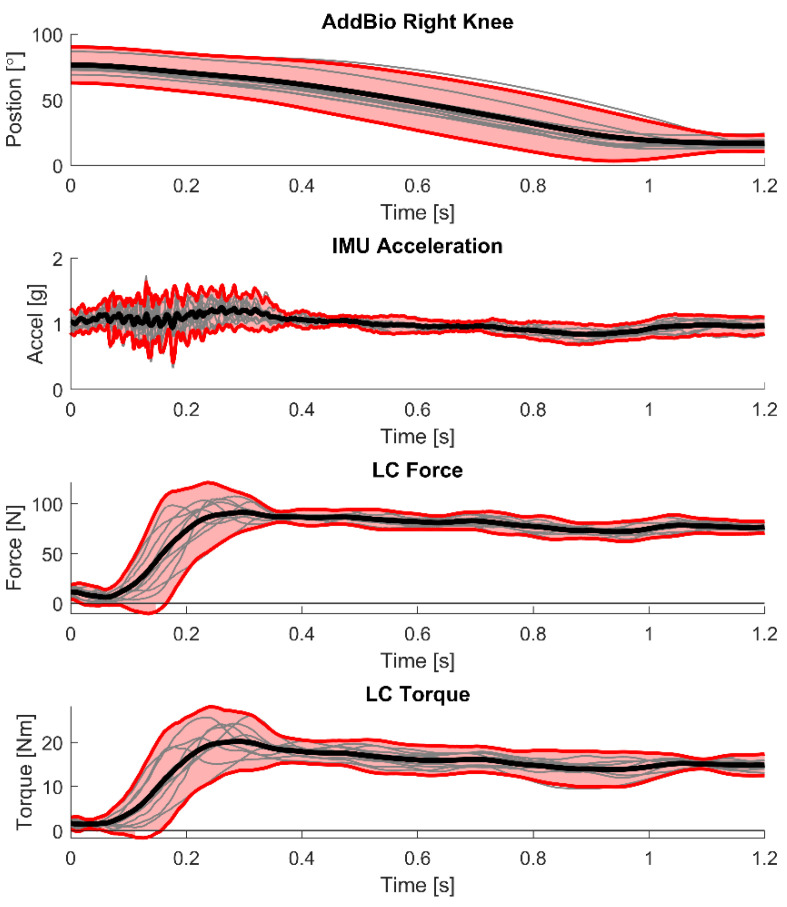
Example of 10 lift cycles of a wheelbarrow from one subject and the sensor readings during lift (IMU, load cell). The gray signals are the 10 trials, the bold red signals are the ±95% confidence intervals, and the black signal is the mean of the signals.

Once the lift cycles are found, the sensor readings from the IMU and load cell are cut and processed based upon the start and end of the lifting cycle to show mean values and confidence intervals during the lifting cycle. The data in Figure 15 show this process applied to knee angle, IMU acceleration, and load cell torque and force. The data shows ten lift cycles of a wheelbarrow from one of the subjects, shown as gray lines in the plot. The larger black signal is the mean value of those signals. The bold red signals represent the ±95% confidence intervals from the data. This analysis shows how this subject typically lifts the wheelbarrow. Future work could then combine data from multiple subjects to show broader trends of how users lift wheelbarrows. The trajectories from different subjects can also be compared using cross-correlation [30] to characterize their similarity.

Once the data are cut up into the ten lifts, the data can be analyzed further. Using the AddBiomechanics data, the angles of the right hip, knee, and ankle are analyzed. The mean and standard deviation of the minimum angle, maximum angle, and the range of motion of the hip, knee, and ankle angles from the 10 lifts are shown in Table 2. This information is useful to understand the amount of movement these joints undergo while lifting a wheelbarrow. These data could be used to compare how different users lift a wheelbarrow in future work.

#### 7.4.2. Gait Characterization: Regular Gait vs. Wheelbarrow Gait

Data can also be analyzed to provide information about gait characteristics. In this case, we show how one subject’s gait varies while pushing the wheelbarrow compared to their regular gait. In the future, spatiotemporal parameters and kinematics data will be examined, and kinetics data will be examined to show nominal parameters during, gait as well as cyclograms, to indicate variations during gait.

Data characterization requires segmenting data based upon the cyclic nature of the data and comparing data between cycles. Data are segmented based upon right heal strike, which is estimated based upon when the right heal marker (i.e., RHEE, Figure 2) z position reaches a minimum. These data are segmented by heel strikes, but midstance and toe off can be used instead following Luttmer et al. midstance and toe-off algorithms in our prior work [31]. Each data segment is then analyzed to estimate spatiotemporal gait parameters for each gait cycle, which then provide mean and standard deviations of the parameters across multiple cycles.

The data library contains six subjects and is growing. An example of spatiotemporal parameters from one subject with ten gait trajectories is shown in Table 3. The table shows the differences in spatiotemporal parameters between the subject’s regular gait and when the subject is manipulating a wheelbarrow while walking. From the table, regular gait step length, stride length, walking speed, cadence, and stride frequency are all larger than that of the subject walking with the wheelbarrow. The step time and stride time is also slower when the subject is manipulating the wheelbarrow, which correlates to the user being more careful while carrying a load. From this analysis, there is a difference in the gait of a subject with and without manipulating an object. Future work will combine all the subjects’ data in the library and perform statistical analysis.

Cyclograms are also used for characterizing gait. Cyclograms plot one joint angle versus another joint angle during a gait cycle [29]. Changes in gait are denoted by changes in the shape of the plots [29]. Comparing knee and hip angle from one leg allows comparison of changes in gait [28,29], while comparing similar joints (e.g., hip or ankle) from the left and right legs can highlight asymmetry in gait [27]. Data provided in this database can provide either type of cyclogram, since the motion of legs was captured.

**Figure 16:**
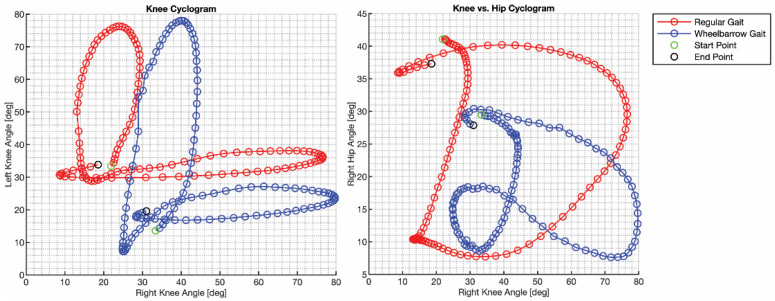
Example cyclogram of a subject’s regular gait versus the subject’s gait while manipulating a wheelbarrow.

This data library contains the regular gait of the subjects and the gait of the subject while walking and interacting with each of the objects. Figure 16 shows the regular gait of a subject compared to the gait of a subject while manipulating a wheelbarrow. The gait is from heel strike to heel strike. The plots on the left side show the knee–knee cyclograms comparing the subject’s regular walking gait and walking gait when pushing a wheelbarrow. The shapes of the plots are similar, but they are offset. When pushing the wheelbarrow, the left leg produces a larger range of motion and the right leg shows a shorter range of motion. The envelopes enclosed by the curves while pushing the wheelbarrow are larger in some regions and smaller in other regions compared to the regular gait, also indicating that there is variation in relative knee angles when pushing the wheelbarrow.

The right subfigure in Figure 16 examines the same walking gaits, but plots the cyclograms comparing the right hip and right knee angle. There is a significant difference between regular walking gait and walking with the wheelbarrow. The range of motion of both the hip and knee have both decreased and shifted. The right knee angle is nominally larger but has less variation. The right hip angle is nominally smaller and shows less variation. As a result, the envelope enclosed by the curve while manipulating the wheelbarrow is significantly smaller compared to the envelope from the regular gait. There is also a small cusp at the bottom left corner of the envelope with regular gait where angles are at their minimum values, whereas the cusp encloses a much larger volume when pushing the wheelbarrow. This indicates that the regular gait joint angles both reach their minimum values simultaneously, whereas the gait with the wheelbarrow results in the joints reaching their minimum in a more staggered behavior. These cyclograms highlight that the gait is very different when the subject is manipulating the object while walking.

## 8. Conclusions

We have presented a database characterizing human interaction with medium-to-large-sized objects from daily living. Unique to this database is its focus on characterizing data important for representing haptic interaction with objects, such as interaction forces, inertial measurements, and object motion, in addition to normal human biomechanical data. Such information should be useful for researchers working in ergonomics, biomechanics, robotics, virtual reality, and gaming.

Methods for collecting the data were presented. The various sensors used to create this database were described. Motion capture marker locations on the human subjects and objects were indicated. The transformations between the sensors, markers, and the human hand were described and included in the library. Data processing through VICON, AddBiomechanics, and MATLAB were presented to produce data that is useful for future applications. An example subset of data was presented to show the output of one of our trials for one object interaction trial. Examples of how these data can be used for different types of processing it were also described. This included looking at ten signals of wheelbarrow lift with means and 95% confidence intervals of the knee joint and sensor signals, accompanied by joint range of motion means and standard deviation of the hip, knee, and ankle. The dataset has gait trials which can be compared, such as the differences between spatiotemporal parameters of a regular gait and when the subject is manipulating a wheelbarrow. Cyclograms of regular gait and gait with a wheelbarrow are shown, which is another way these data can be used. The library is posted on AddBiomechanics, GitHub, and SimTK, with their URLs listed in the Data Availability Statement.

Future work will include continuing to grow the database, identifying object properties, and characterizing human interaction with those objects. Object characteristics will then be programmed into haptic manipulators. Similar studies to those presented here will evaluate human subjects interacting with the manipulators and will be added to this database.

## Figures and Tables

**Table 1:** Comparison of our dataset library and existing ones.

Database	Human Sensing	Haptic Sensing	Object Sensing	Object Size (S, M, L)	Similar Objects	Locomotion with Objects	Number of Subjects
Dataset of Human Motion and Muscular Activities [14]	IMU, sEMG	None	None	M	B	Yes	14
Dataset of Unscripted Human Motion [15]	IMU	None	None	S, M, L	B, D, SC	Yes	13
HBOD [16]	MoCap, IMU	None	MoCap	S, M	None	No	5
An Industry- Oriented Dataset [17]	IMU, MoCap	Pressure Sensor	None	S, M, L	None	Yes	13
BEHAVE [18]	RGBD Images	None	RGBD Images	S, M, L	B, L	No	8
KIT [2]	MoCap	None	MoCap	S, M, L	B	Yes	43
Our Database	MoCap	Load Cell	MocCap, IMU	M, L	B, D, BC, L, W, WB, SC	Yes	6

Box (B), door (D), briefcase (BC), luggage (L), walker (W), wheelbarrow (WB), shopping cart (SC).

**Table 2:** The mean and standard deviation of the minimum, maximum, and range of motion of the knee during a wheelbarrow lift.

Joint	Minimum Angle (Degrees)	Maximum Angle (Degrees)	Range of Motion (Degrees)
Hip	12.5 ± 8.2	75.3 ± 7.4	62.8 ± 7.3
Knee	15.5 ± 3.6	76.6 ± 6.8	61.1 ± 8.2
Ankle	−14.1 ± 1.6	4.3 ± 5.0	18.4 ± 4.2

**Table 3:** Spatiotemporal parameters of a regular gait and gait while manipulating a wheelbarrow.

Parameters	Regular Gait	Wheelbarrow Carry Gait
Step Length [m]	0.66 ± 0.22	0.63 ± 0.03
Step Time [s]	0.58 ± 0.08	0.63 ± 0.04
Stride Length [m]	1.45 ± 0.04	1.24 ± 0.06
Stride Time [s]	1.21 ± 0.03	1.25 ± 0.05
Walking Speed [m/s]	1.20 ± 0.04	0.99 ± 0.06
Cadence [steps/m]	49.7 ± 1.15	48.0 ± 2.00
Stride Frequency [strides/m]	24.9 ± 0.56	24.0 ± 1.00

## Data Availability

Data presented in this library is freely available for public use. Library databases are available at AddBiomechanics, GitHub, and SimTK. The AddBiomechanics URL is: https://addbiomechanics.org/ (accessed on 4 March 2025). The GitHub URL is: https://github.com/nluttmer1/MeLLO-Data-Library (accessed on 4 March 2025). The SimTK URL is: https://simtk.org/projects/mello-library/ (accessed on 4 March 2025) [32].

## References

[1] Feix T., Bullock I.M., Dollar A.M. (2014). Analysis of human grasping behavior: Object characteristics and grasp type. IEEE Trans. Haptics.

[2] Mandery C., Terlemez Ö., Do M., Vahrenkamp N., Asfour T. The KIT whole-body human motion database. Proceedings of the 2015 International Conference on Advanced Robotics (ICAR).

[3] Mandery C., Terlemez Ö., Do M., Vahrenkamp N., Asfour T. (2016). Unifying representations and large-scale whole-body motion databases for studying human motion. IEEE Trans. Robot..

[4] Meixner A., Krebs F., Jaquier N., Asfour T. An Evaluation of Action Segmentation Algorithms on Bimanual Manipulation Datasets. Proceedings of the 2023 IEEE/RSJ International Conference on Intelligent Robots and Systems (IROS).

[5] Kang S., Ishihara K., Sugimoto N., Morimoto J. (2023). Curriculum-based humanoid robot identification using large-scale human motion database. Front. Robot. AI.

[6] Krebs F., Asfour T. (2022). A bimanual manipulation taxonomy. IEEE Robot. Autom. Lett..

[7] Emami M., Bayat A., Tafazolli R., Quddus A. (2024). A survey on haptics: Communication, sensing and feedback. IEEE Commun. Surv. Tutor..

[8] Varalakshmi B., Thriveni J., Venugopal K., Patnaik L. (2012). Haptics: State of the art survey. Int. J. Comput. Sci. Issues.

[9] El Saddik A. (2007). The potential of haptics technologies. IEEE Instrum. Meas. Mag..

[10] Truong T.E., Luttmer N.G., Eshete E.R., Zaki A.B.M., Greer D.D., Hirschi T.J., Stewart B.R., Gregory C.A., Minor M.A. (2022). Evaluating the Effect of Multi-Sensory Stimulation on Startle Response Using the Virtual Reality Locomotion Interface MS.TPAWT. Virtual Worlds.

[11] Russo M., Lee J., Hogan N., Sternad D. (2022). Mechanical effects of canes on standing posture: Beyond perceptual information. J. neuroeng. Rehabil..

[12] McClain E.W. (2022). Simulation and Control of a Human Assistive Quadrupedal Robot. PhD Thesis.

[13] Zhu W., Ma X., Ro D., Ci H., Zhang J., Shi J., Gao F., Tian Q., Wang Y. (2023). Human motion generation: A survey. IEEE Trans. Pattern Anal. Mach. Intell..

[14] Bassani G., Filippeschi A., Avizzano C.A. (2021). A Dataset of Human Motion and Muscular Activities in Manual Material Handling Tasks for Biomechanical and Ergonomic Analyses. IEEE Sens. J..

[15] Geissinger J.H., Asbeck A.T. (2020). Motion inference using sparse inertial sensors, self-supervised learning, and a new dataset of unscripted human motion. Sensors.

[16] Kang P., Zhu K., Jiang S., He B., Shull P. HBOD: A Novel Dataset with Synchronized Hand, Body, and Object Manipulation Data for Human-Robot Interaction. Proceedings of the 2023 IEEE 19th International Conference on Body Sensor Networks (BSN).

[17] Maurice P., Malaisé A., Amiot C., Paris N., Richard G.-J., Rochel O., Ivaldi S. (2019). Human movement and ergonomics: An industry-oriented dataset for collaborative robotics. Int. J. Robot. Res..

[18] Bhatnagar B.L., Xie X., Petrov I.A., Sminchisescu C., Theobalt C., Pons-Moll G. Behave: Dataset and method for tracking human object interactions. Proceedings of the IEEE/CVF Conference on Computer Vision and Pattern Recognition.

[19] Wang Y., Truong T.E., Chesebrough S.W., Willemsen P., Foreman K.B., Merryweather A.S., Hollerbach J.M., Minor M.A. (2020). Augmenting virtual reality terrain display with smart shoe physical rendering: A pilot study. IEEE Trans. Haptics.

[20] Sabetian P. (2019). Modular Cable-Driven Robot Development and Its Applications in Locomotion and Rehabilitation. Ph.D. Thesis.

[21] Duffell L.D., Hope N., McGregor A.H. (2014). Comparison of kinematic and kinetic parameters calculated using a cluster-based model and Vicon’s plug-in gait. Proc. Inst. Mech. Eng. Part H J. Eng. Med..

[22] Goldfarb N., Lewis A., Tacescu A., Fischer G.S. (2021). Open source Vicon Toolkit for motion capture and Gait Analysis. Comput. Methods Programs Biomed..

[23] Hollerbach J.M. Introduction to Robotics: Chapter 7: Velocity and Acceleration. University of Utah. https://my.eng.utah.edu/~cs5310/files/chapter7.pdf.

[24] Werling K., Bianco N.A., Raitor M., Stingel J., Hicks J.L., Collins S.H., Delp S.L., Liu C.K. (2023). AddBiomechanics: Automating model scaling, inverse kinematics, and inverse dynamics from human motion data through sequential optimization. PLoS ONE.

[25] Rajagopal A., Dembia C.L., DeMers M.S., Delp D.D., Hicks J.L., Delp S.L. (2016). Full-body musculoskeletal model for muscle-driven simulation of human gait. IEEE Trans. Biomed. Eng..

[26] Foreman K.B., Wilson C., Dibble L.E., Merryweather A.S. (2019). Training persons with Parkinson disease using an advanced CAVE virtual reality system. FASEB J..

[27] Arippa F., Leban B., Monticone M., Cossu G., Casula C., Pau M. (2022). A study on lower limb asymmetries in Parkinson’s disease during gait assessed through kinematic-derived parameters. Bioengineering.

[28] Chesebrough S.W. (2018). Robot-Assisted Gait Rehabilitation Using Virtual Reality with Torso Force Feedback. Ph.D. Thesis.

[29] Hollerbach J.M., Mills R., Tristano D., Christensen R.R., Thompson W.B., Xu Y. (2001). Torso force feedback realistically simulates slope on treadmill-style locomotion interfaces. Int. J. Robot. Res..

[30] Stoica P., Moses R.L. (2005). Spectral Analysis of Signals.

[31] Luttmer N.G., Truong T.E., Boynton A.M., Carrier D., Minor M.A. Treadmill based three tether parallel robot for evaluating auditory warnings while running. Proceedings of the 2020 IEEE International Conference on Robotics and Automation (ICRA).

[32] Luttmer N.G., Baum N.I., Flores-Gonzalez J., Hollerbach J.M., Minor M.A. MeLLO Data Library (2025-present). GitHub. https://simtk.org/projects/mello-library/.

